# Phytochemical Profile and Biological Activities of the Extracts from Two *Oenanthe* Species (*O. aquatica* and *O. silaifolia*)

**DOI:** 10.3390/ph15010050

**Published:** 2021-12-30

**Authors:** Łukasz Świątek, Elwira Sieniawska, Mohamad Fawzi Mahomoodally, Nabeelah Bibi Sadeer, Krzysztof Kamil Wojtanowski, Barbara Rajtar, Małgorzata Polz-Dacewicz, Mehmet Yavuz Paksoy, Gokhan Zengin

**Affiliations:** 1 Department of Virology with SARS Laboratory, Medical University of Lublin, Chodzki 1, 20-093 Lublin, Poland; barbara.rajtar@umlub.pl (B.R.); malgorzata.polz-dacewicz@umlub.pl (M.P.-D.); 2 Department of Natural Products Chemistry, Medical University of Lublin, Chodzki 1, 20-093 Lublin, Poland; esieniawska@pharmacognosy.org; 3 Department of Health Sciences, Faculty of Medicine and Health Sciences, University of Mauritius, Réduit 80837, Mauritius; f.mahomoodally@uom.ac.mu (M.F.M.); nabsdr15@gmail.com (N.B.S.); 4 Department of Pharmacognosy with Medicinal Plants Garden, Medical University of Lublin, Chodzki 1, 20-093 Lublin, Poland; krzysztofkamilw@gmail.com; 5 Medical Documentation and Secretaryship Programme, Department of Medical Services and Techniques, Tunceli Vocational School, Munzur University, Tunceli 62000, Turkey; mypaksoy@gmail.com; 6 Department of Biology, Science Faculty, Selcuk University, Konya 42130, Turkey

**Keywords:** *Oenanthe*, enzyme inhibition, antiherpetic drugs, oxidative stress, anticancer properties

## Abstract

This study presents the evaluation of biological activities and chemical profiling of *Oenanthe aquatica* (L.) Poir. and *Oenanthe silaifolia* M. Bieb. The phytochemical profile, antioxidant, enzyme inhibitory, cytotoxic and antiviral activities of the methanolic and aqueous extracts were investigated. The aqueous extract of *O. aquatica* possessing the highest content of phenolics (60.85 mg gallic acid equivalent/g extract), also exhibited the strongest radical scavenging potential against 2,2-diphenyl-1-picrylhydrazyl and 2,2′-azino-bis (3-ethylbenzothiazoline-6-sulfonic acid) (79.46 and 148.66 mg Trolox equivalent/g extract, respectively), the highest reducing ability (207.59 and 107.27 mg Trolox equivalent/g extract, for cupric reducing antioxidant capacity and ferric reducing antioxidant activity, respectively), metal chelating potential (33.91 mg ethylenediaminetetraacetic acid equivalent/g extract) and total antioxidant ability (1.60 mmol Trolox equivalent/g extract). Liquid chromatography-electrospray ionization-quadrupole time-of-flight-mass spectrometry (LC-ESI-QTOF-MS/MS) permitted tentative identification of compounds from simple organic acids, phenolic acids, coumarins, flavonoids and their glycosides in *O. aquatica* and *O. silaifolia* extracts. The methanolic extract of *O. aquatica* substantially depressed acetylcholinesterase (3.67 mg galantamine equivalent/g extract), tyrosinase (126.66 mg kojic acid equivalent/g extract), and α-amylase (0.83 mmol acarbose equivalent/g extract) enzymes. The methanolic extract of *O. silaifolia* showed highest enzymatic inhibitory property against butyrylcholinesterase, and its aqueous extract depressed α-glucosidase activity (0.26 mmol acarbose equivalent/g extract). All tested extracts exerted selective toxicity towards cancer cell lines, and the highest anticancer potential was found for *O. aquatica* aqueous extract on FaDu and HeLa cells with CC_50_ of 57.36 and 47.16 µg/mL, respectively. Significant antiviral activity against HSV-1 (HHV-1) was found for both aqueous extracts in concentrations of 1000 µg/mL, which inhibited the HSV-1 cytopathic effect (CPE) in virus infected VERO cells and reduced the virus infective titer by more than 3 log (logCCID_50_/mL). This study has produced critical scientific data on *O. aquatica* and *O. silaifolia,* which are potential contenders for the development of novel phyto-pharmaceuticals.

## 1. Introduction

In our everyday life, the knowledge of traditional medicinal plants is frequently inherited from previous generations and passed down to younger ones. This heirloom of invaluable expertise obtained via ethnobotanical research is essential for conservation and understanding of indigenous and local plant usage [1]. The literature today is rich with growing data emphasizing the necessity of actively screening medicinal plants to disclose crucial information about their therapeutic qualities. Indeed, the widespread growth of the pharmaceutical, nutraceutical, cosmeceutical, and food sectors has resulted in a significant increase in the market for medicinal plants and their bioactive components over time [2].

Research into the phytochemical composition and phytopharmacology of natural products with medicinal properties is an essential stage in evaluating if the product in question shows potential to be successfully introduced into production systems and be used in the pharma industry. Following this line of thought, our aim with this research paper is to screen two medicinally important plants originating from the Apiaceae family and *Oenanthe* genus, namely *Oenanthe aquatica* (L.) Poir. (*O. aquatica*) and *Oenanthe silaifolia* M. Bieb (*O. silaifolia*). *O. aquatica*, also known as fine-leaved water-dropwort, grows mostly in shallow water reservoirs or oxbows with standing water, although it may also be found in field depressions that are temporarily filled with water. It occurs fairly frequently and can develop dense stands, but its communities have a short life period. The morphology of the plant changes in response to changing environmental circumstances [3]. The fruit of *O. aquatica* has antiperiodic, diuretic, expectorant, and pectoral properties. It is used to treat chronic pectoral diseases, dyspepsia, intermittent fevers, persistent ulcers, and other conditions. This plant should be handled with extreme caution and only under the guidance of a knowledgeable practitioner. When consumed in excess, the fruits produce vertigo, drunkenness, and other narcotic symptoms. Externally, the roots have been utilized in pile therapy. The fruits are used to create a homeopathic medicine. It is used to treat bronchitis, coughs, and other respiratory ailments.

In Turkey, the leaves of *O. silaifolia*, also known as narrow leaved water-dropwort, are often eaten raw as a salad or boiled and cooked with rice [4]. However, this *Oenanthe* plant has been linked to livestock poisoning in Greece, causing a central nervous system depressive form of poisoning [5]. We have noticed a shortage of information on the phytopharmacology of *O. aquatica* and *O. silaifolia* in the available literature. Instead, *O. aquatica* species has been studied for its biological or ecological aspects [3], seed reproduction [3], and essential oil [6]. More studies were observed to be conducted on other *Oenanthe* species, notably *O. javanica* (Blume) DC. for its antidiabetic [7], antioxidant and antigenotoxic activities [8,9], anti-hepatitis [10], and phytochemical analysis [11,12].

*O. javanica* is a Chinese traditional medicinal plant and was reported to exert hepatoprotective, anti-inflammatory, immune-modulatory, antioxidant, anticancer, and antiviral properties [10,13,14]. Aerial parts of *O. javanica* were shown to contain biphenyl derivatives exhibiting anti-inflammatory properties by inhibiting cyclooxygenase-2 (COX-2), with 1-(6′-hydroxy-3′-prenyl-phenyl)-10,11-dimethyl-2H-chromen-2-ol possessing the highest activity (IC_50_ 22.18 μM), comparable to celecoxib used as a reference COX-2 inhibitor (IC_50_ 18.08 μM) [15]. Recently, *O. javanica* was reported to display photoprotective activity against UVB-induced collagen disruption and inflammation, which warrants possible application for the treatment of photodamaged skin [16]. Total phenolics isolated from *O. javanica* were shown to possess dose-dependent anti-hepatitis B virus activity, resulting in inhibition of viral antigen production and release in a cell line model (Hep G2.2.15) with inhibition rates of 70.12% (HBeAg) and 72.61% (HBsAg) on day 9. In the duck hepatitis B virus (DHBV) infection model, the total phenolics fraction reduced the DHBV DNA level in a dose-dependent manner [10].

*Oenanthe crocata* essential oil was found to dose-dependently inhibit (IC_50_ 0.36 mg/mL) HIV-1 RT (reverse transcriptase) RDDP (RNA-dependent DNA polymerase)–associated activity. Importantly, even at 16 mg/mL, no inhibition of HIV-1 RT–associated RNase-H activity was found. Unfortunately, the essential oil obtained from *O. crocata* was also proven to significantly decrease cellular viability and fractionation provided fractions which were only weakly active in inhibiting HIV-1 RT RDDP activity [17]. It is also worth mentioning that one of the bioactive constituents in *O. crocata*, oenanthotoxin, is related to cicutoxin and may induce seizures. That is why *O. crocata* (water hemlock dropwort) belongs to the category of highly toxic plant species [18]. There are also reports concerning the presence of potentially toxic substances, such as polyacetylenes (oenanthotoxin) in roots and phenylpropanoids (myristicin) in fruits of *O. aquatica*. These naturally occurring substances may pose a possible health risk for consumers of food products and dietary supplements [19]. Until now, only piecemeal studies on different areas have been conducted on a few *Oenanthe* plants in an unsystematic way while a concise pharmacological validation of these *Oenanthe* members under one single study is clearly missing. Therefore, to fill the research gap, we have attempted to evaluate the antioxidant potential, influence on selected enzymes, anticancer and antiviral activities of *O. aquatica* and *O. silaifolia*, which are two poorly explored *Oenanthe* species. As far as we know, the current literature data on *O. aquatica* and *O. silaifolia* does not provide any information on the influence on clinically important enzymes related to Alzheimer disease (cholinesterases), skin hyperpigmentation (tyrosinase) or diabetes (α-glucosidase and α-amylase). The cytotoxicity of *O. aquatica* and *O. silaifolia* was evaluated towards the normal VERO cell line and a panel of cancer cell lines of various origins (FaDu, HeLa, and RKO). Furthermore, we have evaluated the effect of both extracts on the replication of human herpesvirus type 1 (HSV-1, HHV-1) in infected VERO cells, by the means of cytopathic effect (CPE) reduction, and the decrease of infectious titer using end-point virus titration. We anticipate that the data reported here could bridge a research gap and, as a result, open up new research pathways, notably in the creation of medicinal bioproducts.

## 2. Results and Discussion

### 2.1. Biologically Active Compounds

In Table 1, the total phenolic (TPC) and total flavonoid contents (TFC) of the methanolic and aqueous extracts of *O. aquatica* and *O*. *silaifolia* are shown. In the present work, methanol and water were selected as solvents. In the literature, methanol is considered to be one of the most effective solvents for the extraction of phenolic compounds, especially flavonoids [20,21,22]. Because water has been used to make traditional mixtures of most plants in ethnobotanical work, water was also chosen. Overall, the methanolic extracts yielded the highest TFC while the aqueous extracts yielded the highest TPC. Solvents of higher polarity (water > MeOH) are more effective in recovering phenolic compounds [23]. Herein, the aqueous extracts reported higher TPC in contrast to methanol solvent. However, a different behavior was observed with TFC; methanol was a better extraction solvent in extracting flavonoids. The methanolic and aqueous extracts of *O. aquatica* expressed 42.35 mg RE/g and 60.85 mg GAE/g of TFC and TPC, respectively. The methanolic and aqueous extracts of *O. silaifolia* contained 27.08 mg RE/g and 46.91 mg GAE/g of TFC and TPC, respectively. From the obtained data, it can be said that upon comparison, *O. aquatica* possessed more TFC and TPC than *O. silaifolia*. As far as we know, no previous research has been done on the tested *Oenanthe* species. In agreement with our results, Bhaigyabati et al. [24] reported that the methanolic extract from *O. javanica* contained a higher content of phenolics and flavonoids than the aqueous extract. When comparing our results with previous studies on several *Oenanthe* species, we have also found different results. In a recent study conducted by He et al. [25], the total phenolic content was found to be 199.17 mg GAE/g in the aqueous extract of *O. javanica*, which was higher than that reported in our work. In another study on *O. javanica* [26], the total phenolic content was reported as 44.70 mg GAE/g. Similar content was also reported by Rafat et al. [27], as 38.78 mg GAE/g.

The extracts were analyzed by LC-ESI-QTOF-MS/MS to elucidate the specific profile of *O. aquatica* and *O. silaifolia* extracts. The analysis in negative mode revealed the presence of several dozen compounds belonging to different chemical groups such as simple organic acids, phenolic acids, coumarins, flavonoids and their glycosides. Table 2 shows the differences in the presence of particular compounds in each extract. The highest peak intensity corresponding to the ionization of the molecules was detected for chlorogenic acid, rutin, luteolin, caffeic acid and their derivatives. Only chlorogenic, caffeic and feruoyloquinic, hydroxylinolenic and hydroxylinoleic acids were found in all samples. The exemplary chromatogram is presented in Figure S1. The results of our study are in accordance with literature reports on *Oenanthe* species. The presence of coumarins, phenylpropanoids, and flavonoids in the extracts obtained from aerial parts was previously detected in these species [28,29,30]. Ferulic acid, p-coumaric acid, and 4-hydroxyphenethyl trans-ferulate were isolated from *O. javanica* [29,30]. Methanolic extract of the same species yielded also flavonoids like quercetin, isorhamnetin, nicotiflorin, isoquercitrin, rutin, hyperin, and glycosides [29]. Similarly, *O. fistulosa* was abundant in phenolic acids (15 compounds, including phenylpropanoid skeleton) and flavonoids (17 compounds) [28]. Differences in phytochemical profiles of tested extracts play a significant role in terms of their biological activity and these results should be taken into account in further research on this subject.

### 2.2. Antioxidant Effects

Several recent papers have reported a positive correlation between oxidative stress and the progression of serious health problems [31]. In this sense, antioxidants can alleviate the stress, and they can use various mechanisms such as radical quenching, electron donation or chelation of transition metals. Thus, we used several assays to evaluate antioxidant properties of *O. aquatica* and *O. silaifolia*. The assay results are depicted in Table 1 and Table 3.

Water used for the extraction of antioxidant compounds recorded a higher antioxidant activity in the ABTS^•+^, DPPH, FRAP, CUPRAC, and metal chelating tests in comparison to methanol although in some studies it was the methanolic extracts that showed the strongest activity [32,33,34,35]. Extracts produced using solvents with high polarity often have greater antioxidant activity because the polar phase of the extract encourages the suppression of ABTS^•+^ and DPPH radicals via plain electron and proton relocations [36]. Herein, the aqueous extracts being more polar than methanolic extracts exhibited the highest DPPH and ABTS^•+^ radical scavenging activities. The antioxidant capacity of the extracts was also estimated with regard to its reducing power with the use of CUPRAC and FRAP techniques. Similar to its scavenging abilities, the aqueous extract of *O. aquatica* showed the strongest Cu^2+^ (207.59 mg TE/g) and Fe^3+^ (107.27 mg TE/g) reducing activities.

Plant secondary metabolites have exhibited different antioxidant effects including radical quenching and electron-donation abilities [37]. Similar to DPPH, ABTS^•+^, CUPRAC and FRAP tests, the aqueous extracts (*O. aquatica* and *O. silaifolia*: 33.91 and 28.37 mg EDTAE/g, respectively) showed the most effective chelating ability. However, in terms of total antioxidant capacity, a different behavior was reported. For instance, the methanolic extract of *O. silaifolia* displayed higher activity compared to its aqueous extract. Overall, *O. aquatica* possessed better antioxidant activity than the other *Oenanthe* species, namely *O. silaifolia*, despite originating from the same genus. The reasons why *O. aquatica* has a better potential for antioxidant activity could be, on the one hand, the complication of the effects of ecological influences and, on the other hand, the evolution of divergent biochemical pathways in the affected plants. which has led to the synthesis of diverse antioxidant compounds in *O. aquatica* and not in *O. silaifolia* [38]. Indeed, compounds such as 2-isopropylmalic acid [39], vanillylmandelic acid hexoside [40], hydroxybenzoic acid isomer [41], ethyl syringate hexoside, kaempferol rutinoside [42] and quercetin 7-O-trirhamnoside [42] which were present in *O. aquatica* but found absent in *O. silaifolia*, were reported by different studies to possess good antioxidant activity.

### 2.3. Enzyme Inhibitory Activities

In the present study, the ability of *O. aquatica* and *O. silaifolia* extracts to inhibit the bioactivity of enzymes linked with Alzheimer’s disease (acetylcholinesterase (AChE) and butyrylcholinesterase (BChE)), diabetes type 2 (α-amylase and α-glucosidase), and skin hyperpigmentation (tyrosinase) was tested and the results are shown in Table 4.

Various enzymes in the human organism are involved in the etiology of many diseases, and because of this, inhibiting these enzymes can be a useful method in therapy. Cholinesterase inhibitors, for example, are compounds that prevent the hydrolysis of acetylcholine, a key neurotransmitter for between neural transmission that, when present in lower concentrations, can led to neurodegenerative diseases such as Alzheimer’s and Parkinson’s diseases [43]. In our investigation, we looked for anti-cholinesterase activity in the differently prepared extracts. Relatively similar activity was recorded with both *Oenanthe* species. High anti-AChE and anti-BChE properties were observed in the methanolic extracts while the aqueous extracts were inactive for both plants (*O. aquatica*: AChE = 3.67 and BChE = 5.96 mg GALAE/g; *O. silaifolia*: AChE = 3.35 and BChE = 6.11 mg GALAE/g).

The α-amylase and α-glucosidase inhibitors are important for controlling blood glucose level in diabetes patients [44]. The methanolic extracts of *O. aquatica* and *O. silaifolia* were found to substantially depress α-amylase activity (0.83 and 0.72 mmol ACAE/g, respectively). A different behavior was observed for α-glucosidase inhibitory activity. For instance, the aqueous extract of *O. aquatica* possessed high anti-glucosidase activity (0.26 mmol ACAE/g) while the methanolic extract of *O. silaifolia* exhibited the strongest activity (0.40 mmol ACAE/g).

Tyrosinase inhibitors protect the skin and assist in avoiding hyperpigmentation. The pharmaceutical and cosmeceutical industries strongly support them [45]. The methanolic extracts of *O. aquatica* and *O. silaifolia* displayed the strongest anti-tyrosinase (126.66 and 126.60 mg KAE/g, respectively) activities. It is worth pointing out that the methanolic extracts of both plants exerted the highest potential against four tested enzymes, namely AChE, BChE, tyrosinase and α-amylase, except for α-glucosidase, although the methanolic extracts do not have the highest TFC and TPC. It is possible that there is no crucial relationship between TPC or TFC and enzymatic activities, but that they are more directly connected to other bioactive chemicals. In essence, the biological potential of various phenolic and flavonoid groups varies [46].

Until now, there were no reports on the ability of members of the genus *Oenanthe* to inhibit enzyme activity. In this regard, the given work is the first scientific proof on the enzyme inhibitory actions of *O. aquatica* and *O. silaifolia* extracts, and it may make a significant addition to the scientific platform.

### 2.4. Cytotoxicity Evaluation

The cytotoxicity evaluation of *O. aquatica* and *O. silaifolia* extracts on normal VERO cells showed that the methanolic extracts from both plants show higher toxicity, with CC_50_ values of 340.52 and 252.6 µg/mL (Table 5), respectively, than aqueous extracts, for which the exact CC_50_ values could not be evaluated, but were above 1000 µg/mL. All tested extracts exerted selectivity towards cancer cells, with cells derived from colon carcinoma (RKO) cells being the most resistant ones.

**Figure 1:**
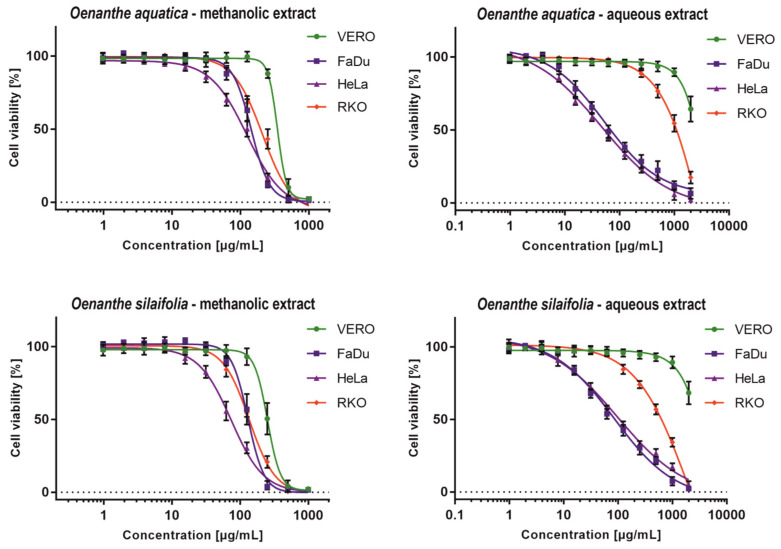
Dose-response influence of *O. aquatica* and *O. silaifolia* extracts on cell lines.

Methanolic extracts exerted significant anticancer potential showing SI between 1.62 and 3.40, with HeLa cells being the most sensitive, especially to *O. silaifolia* methanolic extract (OS-M), which showed CC_50_ of 74.24 µg/mL. Moreover, the OS-M showed the highest toxicity (CC_50_ 135.8 µg/mL) towards RKO amongst all tested extracts. Interestingly, not only were the CC_50_ values obtained for the OS-M on FaDu and RKO cells comparable (Table 5), but also the dose response curves (Figure 1) were similar. Noticeable anticancer activity and selectivity was found for aqueous extracts from *O. aquatica* (OA-A) and *O. silaifolia* (OS-A) on FaDu and HeLa cells, with both cell lines being equally sensitive, as can be concluded from similar dose-response curves (Figure 1) and CC_50_ values (Table 5). The highest anticancer activity and selectivity was found for OA-A on FaDu and HeLa cells with CC_50_ values of 57.36 (SI > 17.43) and 47.16 µg/mL (SI > 21.2), respectively.

The statistical significance of anticancer activity was analyzed using GraphPad Prism software (two-way ANOVA followed by Dunnett’s multiple comparisons test, analyzed with 95% confidence interval). We have compared the CC_50_ values calculated for cancer cell lines with the ones observed for the VERO cells. In case of both methanolic extracts, the differences of CC_50_ values between cancer and normal cells were statistically highly significant (*p* < 0.001). However, because our study design did not allow for accurate measurement of aqueous extracts CC_50_ on VERO cells, we could not subject them to statistical evaluation. Nevertheless, the SI values indicated that aqueous extracts of both species exert anticancer potential, especially towards FaDu and HeLa cells.

To evaluate the cytotoxicity of plant extracts, a classification based on the values of CC_50_ (μg/mL), has been proposed [47]. Taken into account this criterion, all tested extracts can be classified as moderately cytotoxic to FaDu and HeLa cells, whereas, in the case of RKO cells, only *O. silaifolia* methanolic extract can be classified as such. Aqueous extracts showed no cytotoxic activity towards the VERO cells, and methanolic extracts were weakly cytotoxic.

### 2.5. Antiviral Activitiy

**Figure 2:**
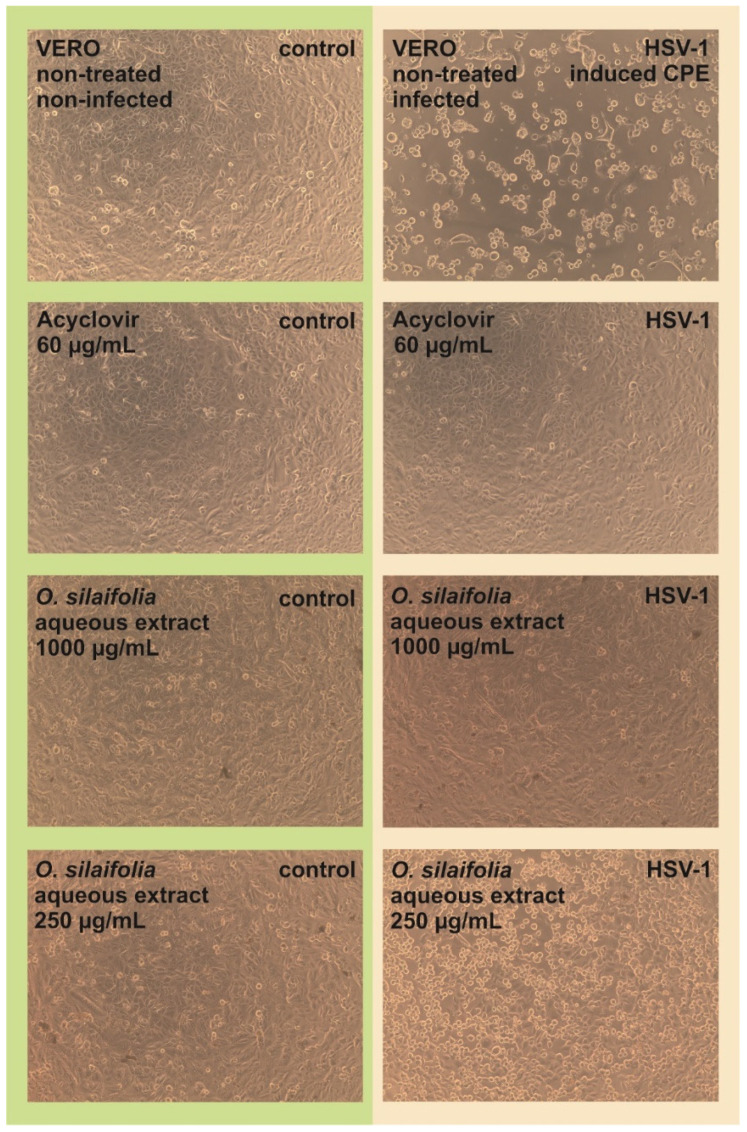
The influence of *O. silaifolia* aqueous extracts on human herpesvirus-1 CPE in VERO cells.

The antiviral activity of *O. aquatica* and *O. silaifolia* extracts was tested on HSV-1 infected VERO cells. Based on the dose-response curves presented in Figure 1, we have selected the highest non-toxic concentrations of OS-M, OA-M to be 150 and 200 µg/mL, and for both aqueous extracts to be 1000 µg/mL. The microscopic observations of the cytopathic effect formation in extract treated HSV-1 infected cells in comparison with untreated infected cells (virus control) showed that in case of both aqueous extracts, the CPE was inhibited, suggesting a potential antiviral effect. The inhibition of CPE formation by OS-A at 1000 µg/mL was presented on Figure 2. Also, *O. aquatica* methanolic extract showed noticeable inhibition of CPE, but not as potent as aqueous extract. Acyclovir was used as a standard antiviral reference substance and completely inhibited CPE formation at 60 µg/mL (Figure 2), whereas a lower dose of 30 µg/mL only partially inhibited the development of CPE.

**Figure 3:**
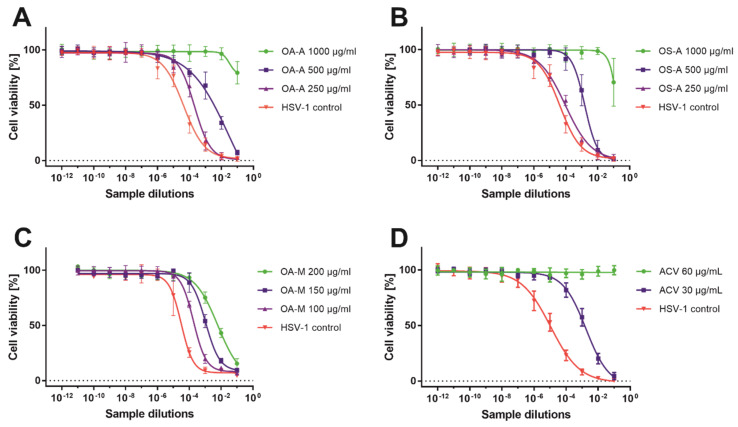
The HSV-1 titration assay of selected samples.

**Figure 4:**
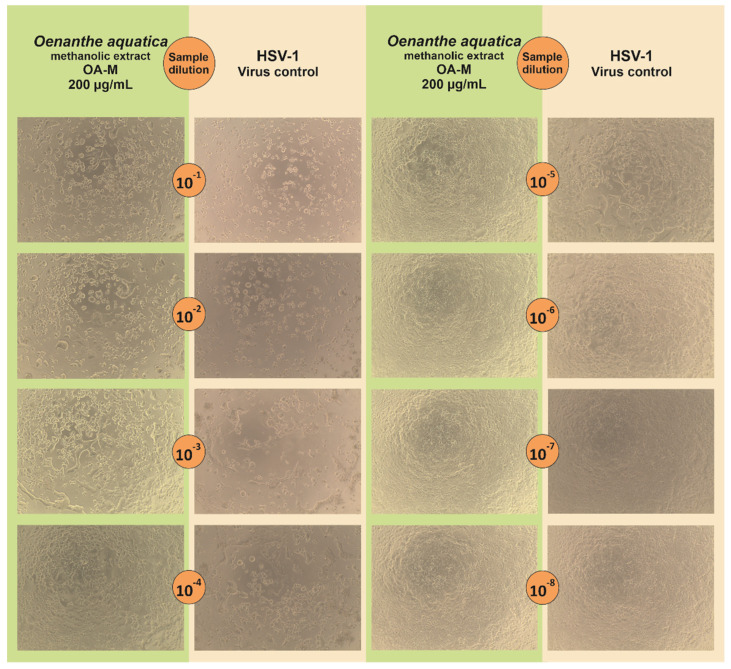
*Oenanthe aquatica* methanolic extract (200 µg/mL) end-point HSV-1 titration assay (the numbers in circles refer to tenfold dilutions of samples collected from antiviral assays).

To evaluate the infectious titer of HSV-1 in collected samples, the end-point titration assay was used and the results are presented in Table 6. The *O. aquatica* methanolic extract showed dose-response antiviral activity (Figure 3C) with the highest decrease of HSV-1 titer (Δlog) of 2.29 at 200 µg/mL (Table 6). Figure 4 shows the end-point HSV-1 titration assay of sample collected from infected VERO cells treated with *O. aquatica* methanolic extract (200 µg/mL) in comparison with the corresponding virus control, confirming the reduction of the infectious titer of HSV-1. In case of *O. silaifolia*, the methanolic extract showed only minor reduction of the virus titer. The acyclovir at 60 µg/mL completely abolished the infectivity of HSV-1 (Figure 3D), whereas the concentration of 30 µg/mL reduced the viral titer by 2.05 log (logCCID_50_/mL) (Table 6). Both aqueous extracts exerted potent reduction of HSV-1 titer at 1000 µg/mL and despite the fact that the accurate amount of viral titer was not calculated (Figure 3A,B), the decrease of the virus titer was no less than 3 log (logCCID_50_/mL). Since a Δlog of at least 3 is required to regard a substance to possess significant antiviral activity, it can be concluded that both aqueous extracts can be classified as such. Herein, the authors would like to point out that the reported antiviral activity of *O. aquatica* and *O. silaifolia* aqueous extracts was observed at relatively high concentration which might be deemed too high for any practical application. However, if future studies allow for separation of bioactive sub-fractions with higher antiviral activity or isolation of compounds responsible for the observed activity, it would greatly benefit the knowledge of natural product derived antivirals. It is still worth mentioning, that this work is the first one to describe anti-HSV activity of *O. aquatica* and *O. silaifolia*.

Organic acids identified in extracts of *O. aquatica* and *O. silaifolia*, namely citric acid (OA-M, OS-A) and malic acid (OA-M, OA-A, OS-A), were shown to exert virucidal activity towards avian influenza virus (AIV, H9N2) [48] and multiple rhinovirus serotypes [49]. However, our study design was focused on the assessment of potential antiviral activity, which is mostly based on the influence of tested substances on intracellular steps of the viral replication cycle, and not on the assessment of virucidal activity.

Caffeic acid and its derivatives identified in *O. aquatica* and *O. silaifolia*, like caffeic acid glucoside, chlorogenic acid, cryptochlorogenic acid, and neochlorogenic acid, were reported to possess antiviral activities towards influenza viruses [50,51,52], human herpesviruses (type-1, type-2), adenoviruses (type -3, -8, and -11) [53], and hepatitis B virus (HBV) [54]. Chlorogenic acid was found to inhibit influenza viruses, strains A/PuertoRico/8/1934(H1N1) (EC_50_ 44.87 μM, EC_50_–50% effective concentration), A/Beijing/32/92(H3N2) (EC_50_ 62.33 μM), as well as strains resistant to oseltamivir; the inhibition occurs during the late stages of the influenza virus infectious cycle, probably due to the inhibition of viral neuraminidase and blocking the release of viral progeny from infected cells [50]. The chlorogenic acid was found to possess antiherpetic (HSV-1, HSV-2), and anti-adenovirus (ADV type-3, -8, and -11) activity, whereas the caffeic acid inhibited both HSV-1 and HSV-2 but only one type of adenovirus (ADV-3) [53]. Interestingly, neochlorogenic acid was reported as a potential anti-SARS-CoV-2 drug, inhibiting the RdRp (RNA-dependent RNA polymerase) of this virus. In silico, neochlorogenic acid interacted with key residues (Arg349, Tyr346 and Phe396) of RdRp [55]. It was also found that the anti-HSV-1 effect of caffeic acid is not because of either the direct HSV-1 inactivation (virucidal effect) or the cytotoxicity of caffeic acid to cells used for virus propagation, but is probably because of the ability to bind to enzymes involved in virus replication cycle [56]. The caffeic acid was found in all tested extracts from both *O. aquatica* and *O. silaifolia* and all extracts showed the ability to decrease the infectious titer of HSV-1; however, only aqueous extracts showed significant antiviral properties. Interestingly, only aqueous extracts showed the presence of neochlorogenic acid and cryptochlorogenic acid (Table 2), and thus we can theorize that those compounds may be responsible for the anti-HSV-1 properties; this requires further studies on isolated compounds. Of course, it is also possible that the synergism of different phenolic acids present in aqueous extracts may contribute to antiviral activity. The methanolic extracts showed higher cytotoxicity to VERO cells which resulted in lower concentrations used in antiviral assays and possibly also contributed to lower antiviral effects.

## 3. Materials and Methods

### 3.1. Plant Material and Extraction Procedure

*Oenanthe* species were collected in July 2020 from Turkey (*O. silaifolia* M. Bieb.: Tunceli, Mazgirt, Yukarı Oyumca village, 1400 m; *O. aquatica* (L.) Poir.: Edirne; Enez, Gala Lake, 150 m). Voucher specimens were deposited in Munzur University (voucher numbers: Paksoy 1397 and Paksoy 1187, respectively). The aerial parts were dried in a well-ventilated place for ten days and then ground using one laboratory mill.

For each species, methanol and water were selected as solvents. In the preparation of methanol extracts, the plant materials (5 g) were extracted at room temperature with 100 mL of methanol for 24 h. Then, the extracts were filtered, and the solvents were removed by using a rotary-evaporator. As for the water extracts, the plant samples (5 g) were extracted with 100 mL of boiled water for 15 min. Then, the mixtures were filtered and lyophilized. The obtained extracts were kept at 4 °C before analysis. The extraction yields were given in Table 1.

### 3.2. Total Content of Phenolics and Flavonoids

Total phenolic content (TPC) and total flavonoid content (TFC) were assessed according to previously described methods [57,58]. TPC was expressed as mg gallic acid equivalents (GAE)/g dry extract, whereas TFC was calculated as mg rutin equivalents (RE)/g dry extract. All experimental details of the assays are given in Supplemental Material.

### 3.3. Antioxidant Properties and Enzyme Inhibition

In the current work, the antioxidant effects of the tested extracts were detected by different assays [57]. The assays were [1,1-diphenyl-2-picrylhydrazyl (DPPH) and 2,2′-azino-bis(3-ethylbenzothiazoline) 6-sulfonic acid (ABTS) radical scavenging, cupric ion reducing antioxidant capacity (CUPRAC), ferric ion reducing antioxidant power (FRAP), metal chelating ability (MCA) and phosphomolybdenum assay (PDA)]. For DPPH, ABTS, CUPRAC and FRAP assay data were expressed as mg Trolox equivalents (TE)/g extract, whereas in MCA and PDA, mg EDTA equivalents (EDTAE)/g extract and mmol TE/g extract, respectively, were used. The experimental details for acetylcholinesterase, butyrylcholinesterase, tyrosinase, amylase and glucosidase assays were previously provided. Galathamine was used as a positive control in cholinesterase assays and data were evaluated as mg galanthamine equivalents (GALAE)/g extract. Kojic acid was used as a standard inhibitor in tyrosinase inhibitory assay and the results were expressed as mg kojic acid equivalents (KAE)/g extract [57,58]. Acarbose was selected as inhibitor for amylase and glucosidase inhibitory assays and the results are given as mmol acarbose equivalents (ACAE)/g extract. All experimental details of the assays are given in supplemental material. The assays were performed in triplicate and the differences in the extracts were evaluated by ANOVA assays (Tukey’s test).

### 3.4. LC-ESI-QTOF-MS/MS Analysis

The constituents of the studied extracts were separated on the on Gemini^®^ column (3 μm i.d. C18 with TMS endcapping, 110 Å, 100 × 2 mm) guarded with pre-column (Phenomenex Inc, Torrance, CA, USA) following chromatographic conditions previously described [59]. The separation was performed on Agilent 1200 Infinity HPLC (Agilent Technologies, Santa Clara, CA, USA), whereas detection was obtained on Agilent 6530B QTOF (Agilent Technologies, Santa Clara, CA, USA). 2 mass spectra/s were registered in a scan range 100–1700 *m*/*z* for MS and MS/MS, applying collision energy of 10 and 40 eV. The drying gas temperature and flow were set at 300 °C and 12 L/min, whereas the sheath gas temperature and flow were set at 325 °C and 12 L/min, respectively. The ion source operated in negative mode with 40 psig, capillary V (+): 4000 V, skimmer 65 V. Freely available mass spectra databases (MassBank, PubChem, HMDB, Metlin, MoNA) were used for tentative identification of compounds, which was also supported by fragmentation patterns published in scientific literature.

### 3.5. Cell Assays

Media used for in vitro culturing included Dulbecco Modified Eagle Medium (DMEM, Corning, Tewksbury, MA, USA) used for VERO cells, and Modified Eagle Medium (MEM, Corning) used for other cell lines. Cell media used in the experiments were supplemented with antibiotics and fetal bovine serum. Incubation was carried out in a CO_2_ incubator (5% CO_2_, 37 °C). Detailed description of cell line maintenance can be found in the Supplementary Materials.

*O. aquatica* and *O. silaifolia* methanolic extracts were dissolved in DMSO, and aqueous extracts in PBS. Stock solutions (50 mg/mL) were membrane filtered (0.2 µm) and stored frozen (−20 °C) until used.

### 3.6. Cytotoxicity Assessment

Cytotoxicity was tested using MTT based assay following a previously described protocol [60]. Briefly, the monolayers of the appropriate cell lines in 96-well plates were incubated for 72 h with serial dilutions of extract stock solutions (methanolic extracts: 1000–0.98 µg/mL, aqueous extracts: 2000–0.98 µg/mL). Afterwards, the MTT assay was used for the evaluation of cellular viability and the absorbance was measured using a microplate reader. Collected data was analyzed with GraphPad Prism software and the values of CC_50_ (the 50% cytotoxic concentration–concentration resulting in 50% reduction of cell viability) were calculated from dose-response curves (non-linear regression). Moreover, the selectivity towards cancer cells was assessed by calculating the selectivity indexes (VERO CC_50_/cancer cell line CC_50_). In additional, CC_10_ (10% cytotoxic concentration–concentration resulting in 10% reduction of cell viability) values of tested extracts towards VERO cells were calculated for use in antiviral studies. Detailed descriptions of cytotoxicity testing can be found in the Supplementary Materials.

### 3.7. Antiherpetic Assay and the Evaluation of HSV-1 Titer

Antiviral activity was evaluated towards human herpesvirus-1 (HSV-1) replicating in VERO cells, as previously described [60]. The infectious titer of HSV-1 used in this study was 5.5 ± 0.25 logCCID_50_/mL (CCID_50_–50% cell culture infectious dose). This assay is based on the evaluation of the effect of tested extract on the formation of the cytopathic effect in virus infected cell line (after 1 h preincubation with 100-fold CCID_50_ of HSV-1). Extracts showing antiviral activity should inhibit the CPE.

Samples collected from antiviral assays were further subjected to end-point dilution assay to evaluate HSV-1 titers. Briefly, the VERO cells (monolayer) in 96-well plates were incubated with ten-fold dilutions of samples (3 replicates) in cell media for 72 h. Daily observation was conducted to monitor the development of CPE during titration. Subsequently, the MTT test was used to evaluate CCID_50_ and the values obtained for tested extracts were compared with the titer of non-treated infected cells. The detailed methodology can be found in the Supplementary Materials. In order to report that an extract significantly inhibits viral replication it should reduce the CCID_50_ by ≥3 log.

### 3.8. Data Analysis

The GraphPad Prism software was used for the evaluation of statistical significance of obtained data (two-way ANOVA with Dunnett’s multiple comparisons test).

## 4. Conclusions

The investigation of *O. aquatica* and *O. silaifolia* bio components described in this study adds to our understanding of the phytochemistry of these plants and enhances our understanding of their antioxidant, enzyme inhibitory properties, cytotoxicity, and antiviral activities. Aqueous extract of both plants showed the most potent antioxidant properties, with most of tests indicating correlation with their significant TPC. The methanolic extract of *O. aquatica* depressed the inhibitory activities of most enzymes including AChE, tyrosinase, and α-amylase. All tested extracts exerted noticeable anticancer activity towards hypopharyngeal squamous cell carcinoma (FaDu) and cervical adenocarcinoma (HeLa), whereas colon carcinoma derived cells (RKO) proved to be more resistant. The data presented herein demonstrate that neochlorogenic acid and cryptochlorogenic acid present in only aqueous extracts may be responsible for the anti-HSV-1 properties. However, further study, including clinical in vivo investigations, is needed to examine these aforementioned characteristics in order to incorporate these traditional plants as possible pharmaceutical components.

## Figures and Tables

**Table 1:** Extraction yields, amount of biologically active substances and total antioxidant capacity (by PDA assays) of the analyzed extracts.

Samples	Extraction Yields (%)	TPC (mg GAE/g)	TFC (mg RE/g)	PBD (mmol TE/g)
*O. aquatica*-MeOH	6.88	39.05 ± 0.76 ^c^	42.35 ± 2.16 ^a^	1.38 ± 0.16 ^ab^
*O. aquatica*-Water	10.39	60.85 ± 0.38 ^a^	14.84 ± 0.06 ^c^	1.60 ± 0.13 ^a^
*O. silaifolia*-MeOH	6.82	36.76 ± 0.05 ^d^	27.08 ± 0.70 ^b^	1.35 ± 0.12 ^ab^
*O. silaifolia*-Water	8.74	46.91 ± 0.32 ^b^	11.58 ± 0.15 ^d^	1.27 ± 0.07 ^b^

Values are reported as mean ± SD. MeOH: Methanol; TPC: Total phenolic content; TFC: Total flavonoid content; PBD: Phosphomolybdenum; GAE: Gallic acid equivalent; RE: Rutin equivalent; TE: Trolox equivalent. Different superscripts indicate significant differences in the tested extracts (*p* < 0.05).

**Table 2:** The chemical composition of studied extracts.

No	Retention Time [Min]	Name	Formula	Molecular Ion [M-H]^−^	Fragmentation Ions	*O. aquatica*-MeOH	*O. aquatica*-Water	*O. silaifolia*-MeOH	*O. silaifolia*-Water
1	1.969	Caffeic acid hexoside derivative	C_15_H_18_O_9_	377.0901	341.1129; 215.0327; 179.0637	+	−	+	−
2	1.921	Malic acid	C_4_H_6_O_5_	133.0161	115.0014; 71.0123	+	+	−	+
3	1.266	Citric acid	C_6_H_8_O_7_	191.0218	129.0157; 111.0072	+	−	−	+
4	7.476	Dihydroxybenzoic acid	C_7_H_6_O_4_	153.0208	109.0302; 108.0203; 91.0152; 53.0379	+	+	+	−
5	8.092	Hydroxybenzoic acid glucoside	C_7_H_6_O_3_	299.0816	137.0260; 119.0373	+	−	−	−
6	8.390	2-Isopropylmalic acid	C_7_H_12_O_5_	175.0613	115.0413; 85.0679	+	+	−	−
7	8.927	Vanillylmandelic acid hexoside	C_15_H_20_O_10_	359.1019	197.0473; 153.0544; 138.0293	+	+	−	−
8	9.531	Hydroxybenzoic acid	C_7_H_6_O_3_	137.0267	108.0221	+	+	+	−
9	9.562	Neochlorogenic acid	C_16_H_18_O_9_	353.0918	191.0630; 179.0335; 135.0439	−	+	−	+
10	10.376	Caffeic acid glucoside	C_15_H_18_O_9_	341.0955	179.0382; 161.0231	+	+	+	−
11	10.751	Aesculin	C_15_H_16_O_9_	339.0763	177.0214; 133.0305	+	+	+	−
12	14.506	Hydroxybenzoic acid isomer	C_7_H_6_O_3_	137.0263	108.0191	+	+	−	−
13	15.120	Aesculetin	C_9_H_6_O_4_	177.0226	133.0311; 105.0374	+	−	+	−
14	15.487	Chlorogenic acid	C_16_H_18_O_9_	353.0929	191.0596	+	+	+	+
15	16.147	Cryptochlorogenic acid	C_16_H_18_O_9_	353.0885	191.0569; 179.0364; 173.0466; 135.0435	−	+	−	+
16	16.312	Caffeic acid	C_9_H_8_O_4_	179.0381	135.0462; 107.0457	+	+	+	+
17	19.856	Feruoyloquinic acid	C_17_H_20_O_9_	367.1081	191.0575; 173.0467	+	+	+	+
18	20.683	Ethyl syringate hexoside	C17H24O10	387.1356	225.0788; 210.0539; 180.0449	+	−	−	−
19	21.640	Unknown		467.1638	241.0053; 996.9607	+	+	+	+
20	22.875	Caffeic acid derivative hexoside		365.0554	203.0300; 185.0198; 179.0407; 141.0227; 135.0477	+	+	+	+
21	23.380	Rutin	C_27_H_30_O_16_	609.1517	300.0251; 271.0206; 255.0227; 179.0006; 150.9982	+	+	+	−
22	24.105	Isoquercetin	C_21_H_20_O_12_	463.0928	300.0268; 271.0233; 255.0312; 151.0060	+	+	+	−
23	25.108	Kaempferol rutinoside	C_27_H_30_O_15_	593.1565	285.0431; 255.0307; 229.0351	+	+	−	−
24	25.754	3-*O*-rhamnetin rutinoside	C_28_H_32_O_16_	623.1666	315.0511	+	−	+	+
25	26.547	Dicaffeoyloquinic acid	C_22_H_28_O_14_	515.1206	353.0935; 191.0560; 179.0351; 173.0452	+	+	+	−
26	27.190	Luteolin derivative		635.1667	285.0370	+	−	+	−
27	29.912	Unknown		449.1527	363.0728; 241.0030; 96.9611	+	+	+	+
28	32.700	Caffeic acid methyl ester derivative	C_11_H_22_O_11_	329.1074	193.1355; 179.0328; 161.0244; 135.0466	+	+	+	−
29	34.487	Luteolin	C_15_H_10_O_6_	285.0443	133.0236; 117.0339	+	−	+	−
30	34.657	3-*O*-methyl quercetin	C_16_H_12_O_7_	315.0551	300.0262; 271.0323; 255.0307; 151.0014; 108.0244	+	−	+	−
31	41.611	Quercetin 7-*O*-trirhamnoside	C_32_H_36_O_20_	739.1740	593.1260; 301.0337; 271.0268; 179.9964; 151.0032	+	−	−	−
32	46.525	Hydroxylinolenic acid	C_18_H_30_O_3_	293.2109	275.2109; 171.1001; 121.0979	+	+	+	+
33	49.117	Hydroxylinoleic acid	C_18_H_32_O_3_	295.2324	277.2170; 171.1023; 123.1179	+	+	+	+

+: present; −: absent.

**Table 3:** Antioxidant activity of the analyzed extracts.

Samples	DPPH (mg TE/g)	ABTS (mg TE/g)	CUPRAC (mg TE/g)	FRAP (mg TE/g)	MCA (mg EDTAE/g)
*O. aquatica*-MeOH	50.58 ± 1.03 ^c^	74.15 ± 1.74 ^c^	147.08 ± 7.62 ^b^	73.65 ± 0.26 ^c^	16.46 ± 0.56 ^c^
*O. aquatica*-Water	79.46 ± 0.40 ^a^	148.66 ± 2.17 ^a^	207.59 ± 1.82 ^a^	107.27 ± 0.55 ^a^	33.91 ± 0.84 ^a^
*O. silaifolia*-MeOH	39.07 ± 0.98 ^d^	77.55 ± 2.94 ^c^	88.62 ± 2.01 ^c^	62.04 ± 0.66 ^d^	11.15 ± 0.60 ^d^
*O. silaifolia*-Water	66.34 ± 3.49 ^b^	118.28 ± 0.53 ^b^	155.19 ± 2.24 ^b^	83.02 ± 0.58 ^b^	28.37 ± 0.83 ^b^

Values are shown as mean ± SD. MeOH: Methanol; TE: Trolox equivalent; EDTAE: EDTA equivalents. Different superscripts indicate significant differences in the tested extracts (*p* < 0.05).

**Table 4:** Inhibition of selected enzymes exerted by the analyzed extracts.

Samples	AChE (mg GALAE/g)	BChE (mg GALAE/g)	Tyrosinase (mg KAE/g)	Amylase (mmol ACAE/g)	Glucosidase (mmol ACAE/g)
*O. aquatica*-MeOH	3.67 ± 0.15 ^a^	5.96 ± 0.52 ^a^	126.66 ± 0.95 ^a^	0.83 ± 0.02 ^a^	0.16 ± 0.04 ^c^
*O. aquatica*-Water	na	na	6.31 ± 0.81 ^b^	0.15 ± 0.01 ^c^	0.26 ± 0.03 ^bc^
*O. silaifolia*-MeOH	3.35 ± 019 ^b^	6.11 ± 0.41 ^a^	126.60 ± 1.88 ^a^	0.72 ± 0.03 ^b^	0.40 ± 0.06 ^a^
*O. silaifolia*-Water	na	na	4.82 ± 0.17 ^b^	0.13 ± 0.01 ^c^	0.28 ± 0.03 ^b^

Values are shown as mean ± SD. MeOH: Methanol; GALAE: Galantamine equivalent; KAE: Kojic acid equivalent; ACAE: Acarbose equivalent; na: not active. Different superscripts indicate significant differences in the tested extracts (*p* < 0.05).

**Table 5:** Cytotoxicity of *O. aquatica* and *O. silaifolia* extracts.

Plant	Solvent–Sample	VERO	FaDu		HeLa		RKO	
		CC_50_ *	CC_50_	SI **	CC_50_	SI	CC_50_	SI
*Oenanthe aquatica*	methanol–OA-M	340.52 ± 22.83	142.13 ± 10.46	2.40	123.47 ± 14.35	2.76	209.73 ± 17.84	1.62
	water–OA-A	>1000	57.36 ± 7.21	>17.43	47.16 ± 3.44	>21.2	1001.47 ± 63.84	>1
*Oenanthe silaifolia*	methanol–OS-M	252.6 ± 32.05	129.33 ± 15.27	1.95	74.24 ± 9.48	3.40	135.8 ± 4.11	1.86
	water–OS-A	>1000	90.35 ± 5.08	>11.07	101.31 ± 22.82	>9.87	552.73 ± 37.56	>1.81

* mean ± SD (µg/mL); CC_50_—the concentration decreasing viability by 50%; ** SI—the selectivity index (CC_50_VERO/CC_50_ Cancer Cells.

**Table 6:** Decrease of HSV-1 titer by the *O. aquatica* and *O. silaifolia* extracts.

Substance	Solvent [Sample]	Concentration (µg/mL)	Reduction of HSV-1 Infectious Titer (Δlog) *
*Oenanthe aquatica*	methanol [OA-M]	200	2.29 ± 0.46
		150	1.56 ± 0.13
		100	0.73 ± 0.1
	water [OA-A]	1000	>3
		500	2.05 ± 0.35
		250	0.57 ± 0.23
*Oenanthe silaifolia*	methanol [OS-M]	150	1.1 ± 0.33
		100	0.38 ± 0.08
	water [OA-A]	1000	>3
		500	1.81 ± 0.26
		250	0.3 ± 0.05
Acyclovir	n/a [ACV]	60	>3
		30	2.05 ± 0.35

* Δlog (mean ± SD)—evaluated from end-point titration performed using samples collected from independent antiviral assays. Δlog = logCCID_50_HC—logCCID_50_OE; HC—herpesvirus control; OE—*Oenanthe* extract, significant antiviral activity ≥ 3 log reduction of HSV-1 titer; n/a—not applicable.

## Data Availability

Not applicable.

## Supplementary Materials

The following are available online at https://www.mdpi.com/article/10.3390/ph15010050/s1, Figure S1: The BPC chromatogram of *O. aquatica*-MeOH; numbers correspond to Table 2.
