# Supplementary material for: Phytochemical Profile and Biological Activities of the Extracts from Two Oenanthe Species (O. aquatica and O. silaifolia)
Source: Pharmaceuticals (Basel). 2021 Dec 30;15(1):50. doi: 10.3390/ph15010050 (PMC8779323; doi:10.3390/ph15010050)
Supplement: Supplementary file 1 [file pharmaceuticals-15-00050-s001.zip › pharmaceuticals-1424102 - Supplementary Material.pdf]

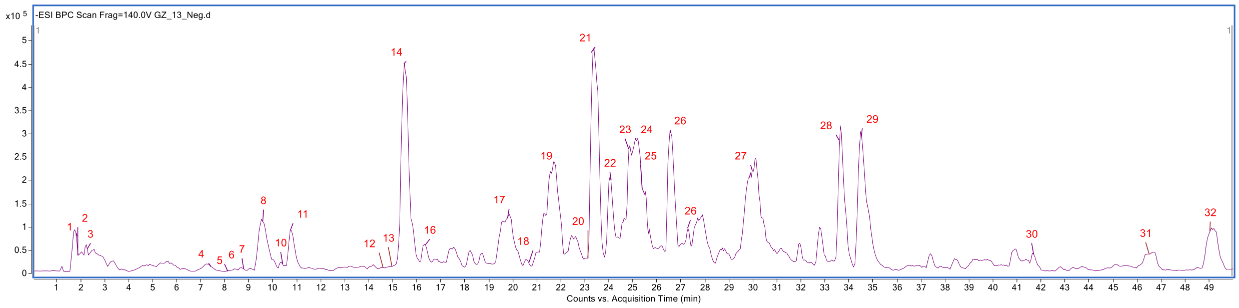

Figure S1. The BPC chromatogram of *O. aquatica*-MeOH; numbers correspond to Table 2.

Assays for Total Phenolic and Flavonoid Contents

The total phenolic content was determined by employing the methods given in the literature with some modification. Sample solution (2 mg/mL; 0.25 mL) was mixed with diluted Folin–Ciocalteu reagent (1 mL, 1:9, *v*/*v*) and shaken vigorously. After 3 min, Na_2CO_3 solution (0.75 mL, 1%) was added and the sample absorbance was read at 760 nm after a 2 h incubation at room temperature. The total phenolic content was expressed as milligrams of gallic acid equivalents (mg GAE/g extract)

The total flavonoid content was determined using the $AlCl_3$ method. Briefly, sample solution (2mg/mL; 1 mL) was mixed with the same volume of aluminum trichloride (2%) in methanol. Similarly, a blank was prepared by adding sample solution (1 mL) to methanol (1 mL) without $AlCl_3$. The sample and blank absorbances were read at 415 nm after a 10 min incubation at room temperature. The absorbance of the blank was subtracted from that of the sample. Rutin was used as a reference standard and the total flavonoid content was expressed as milligrams of rutin equivalents (mg RE/g extract)

Determination of Antioxidant and Enzyme Inhibitory Effects

For the DPPH (1,1-diphenyl-2-picrylhydrazyl) radical scavenging assay: Sample solution (2mg/ml, 1 mL) was added to 4 mL of a 0.004% methanol solution of DPPH. The sample absorbance was read at 517 nm after a 30 min incubation at room temperature in the dark. DPPH radical scavenging activity was expressed as milligrams of trolox equivalents (mg TE/g extract).

For ABTS (2,2′-azino-bis(3-ethylbenzothiazoline) 6-sulfonic acid) radical scavenging assay: Briefly, ABTS+ was produced directly by reacting 7 mM ABTS solution with 2.45 mM potassium persulfate and allowing the mixture to stand for 12–16 in the dark at room temperature. Prior to beginning the assay, ABTS solution was diluted with methanol to an absorbance of 0.700 ± 0.02 at 734 nm. Sample solution (2 mg/mL, 1 mL) was added to ABTS solution (2 mL) and mixed. The sample absorbance was read at 734 nm after a 30 min incubation at room temperature. The ABTS radical scavenging activity was expressed as milligrams of trolox equivalents (mg TE/g extract).

For CUPRAC (cupric ion reducing activity) activity assay: Sample solution was added to premixed reaction mixture containing $CuCl_2$ (1 mL, 10 mM), neocuproine (1 mL, 7.5 mM) and NH_4Ac buffer (1 mL, 1 M, pH 7.0). Similarly, a blank was prepared by adding sample solution (2 mg/mL; 0.5 mL) to premixed reaction mixture (3 mL) without $CuCl_2$. Then, the sample and blank absorbances were read at 450 nm after a 30 min incubation at room temperature. The absorbance of the blank was subtracted from that of the sample. CUPRAC activity was expressed as milligrams of trolox equivalents (mg TE/g extract).

For FRAP (ferric reducing antioxidant power) activity assay: Sample solution (2mg/mL; 1 mL) was added to premixed FRAP reagent (2 mL) containing acetate buffer (0.3 M, pH 3.6), 2,4,6-tris(2-pyridyl)-S-triazine (TPTZ) (10 mM) in 40 mM HCl and ferric chloride (20 mM) in a ratio of 10:1:1 (*v*/*v*/*v*). Then, the sample absorbance was read at 593 nm after a 30 min incubation at room temperature. FRAP activity was expressed as milligrams of trolox equivalents (mg TE/g extract).

For phosphomolybdenum method: Sample solution (2 mg/ml, 1 mL) was combined with 3 mL of reagent solution (0.6 M sulfuric acid, 28 mM sodium phosphate and 4 mM ammonium molybdate). The sample absorbance was read at 695 nm after a 90 min incubation at 95 °C. The total antioxidant capacity was expressed as millimoles of trolox equivalents (mmol TE/g extract).

For metal chelating activity assay: Briefly, sample solution (2 mg/mL, 1 mL) was added to $FeCl_2$ solution (0.05 mL, 2 mM). The reaction was initiated by the addition of 5 mM ferrozine (0.2 mL). Similarly, a blank was prepared by adding sample solution (2 mL) to $FeCl_2$ solution (0.05 mL, 2 mM) and water (0.2 mL) without ferrozine. Then, the sample and blank absorbances were read at 562 nm after 10 min incubation at room temperature. The absorbance of the blank was sub-tracted from that of the sample. The metal chelating activity was expressed as milligrams of EDTA (disodium edetate) equivalents (mg EDTAE/g extract).

For Cholinesterase (ChE) inhibitory activity assay: Sample solution (2 mg/mL, 100 μL)was mixed with DTNB (5,5-dithio-bis(2-nitrobenzoic) acid, Sigma, St. Louis, MO, USA) (125 μL) and AChE (acetylcholines-terase (Electric ell acetylcholinesterase, Type-VI-S, EC 3.1.1.7,Sigma)), or BChE (butyrylcholinesterase (horse serum butyrylcholinesterase, EC 3.1.1.8, Sigma)) solution (25 μL) in Tris–HCl buffer (pH 8.0) in a 96-well microplate and incubated for 15 min at 25 °C. The reaction was then initiated with the addition of acetylthiocholine iodide (ATCI, Sigma) or butyrylthiocholine chloride (BTCl, Sigma) (25 μL). Similarly, a blank was prepared by adding sample solution to all reaction reagents without enzyme (AChE or BChE) solution. The sample and blank absorbances were read at 405 nm after 10 min incubation at 25 °C. The absorbance of the blank was subtracted from that of the sample and the cholinesterase inhibitory activity was expressed as galanthamine equivalents (mgGALAE/g extract).

For Tyrosinase inhibitory activity assay: Sample solution (2 mg/ml, 50 μL) was mixed with tyrosinase solution (40 μL, Sigma) and phosphate buffer (100 μL, pH 6.8) in a 96-well microplate and incubated for 15 min at 25 °C. The reaction was then initiated with the addition of L-DOPA (40 μL, Sigma). Similarly, a blank was prepared by adding sample solution to all reaction reagents without enzyme (tyrosinase) solution. The sample and blank absorbances were read at 492 nm after a 10 min incubation at 25 °C. The absorbance of the blank was subtracted from that of the sample and the tyrosinase inhibitory activity was expressed as kojic acid equivalents (mgKAE/g extract).

For α-amylase inhibitory activity assay: Sample solution (2 mg/ml, 50 μL) was mixed with α-amylase solution (ex-porcine pancreas, EC 3.2.1.1, Sigma) (50 μL) in phosphate buffer (pH 6.9 with 6 mM sodium chloride) in a 96-well microplate and incubated for 10 min at 37 °C. After pre-incubation, the reaction was initiated with the addition of starch solution (50 μL, 0.05%). Similarly, a blank was prepared by adding sample solution to all reaction reagents without enzyme (α-amylase) solution. The reaction mixture was incubated 10 min at 37 °C. The reaction was then stopped with the addition of HCl (25 μL, 1 M). This was followed by addition of the iodine-potassium iodide solution (100 μL). The sample and blank absorbances were read at 630 nm. The absorbance of the blank was subtracted from that of the sample and the α-amylase inhibitory activity was expressed as acarbose equivalents (mmol ACE/g extract).

For α-glucosidase inhibitory activity assay: Sample solution (2 mg/ml, 50 μL) was mixed with glutathione (50 μL), α-glucosidase solution (from Saccharomyces cerevisiae, EC 3.2.1.20, Sigma) (50 μL) in phosphate buffer (pH 6.8) and PNPG

(4-N-trophenyl-α-D-glucopyranoside, Sigma) (50 μL) in a 96-well microplate and incubated for 15 min at 37 °C. Similarly, a blank was prepared by adding sample solution to all reaction reagents without enzyme (α-glucosidase) solution. The reaction was then stopped with the addition of sodium carbonate (50 μL, 0.2 M). The sample and blank absorbances were read at 400 nm. The absorbance of the blank was subtracted from that of the sample and the α-glucosidase inhibitory activity was expressed as acarbose equivalents (mmol ACE/g extract).

In Vitro Studies

The cytotoxicity of *O. aquatica* and *O. silaifolia* was evaluated *in vitro* towards normal VERO (ATCC, Cat. No. CCL-81) cells and cancer derived cell lines – FaDu (ATCC, Cat. No. HTB-43, hypopharyngeal squamous cell carcinoma), HeLa (ECACC, Cat. No. 93021013, cervical adenocarcinoma) and RKO (ATCC, Cat. No. CRL-2577, colon carcinoma), using 3-(4,5-dimethylthiazol-2-yl)-2,5-diphenyltetrazolium bromide (MTT) based protocol.

Media used for in vitro culturing included Dulbecco Modified Eagle Medium (DMEM, Corning, Tewksbury, MA, USA) used for VERO cells, and Modified Eagle Medium (MEM, Corning) used for other cell lines. Cell media used in the experiments were supplemented with antibiotics (Penicillin-Streptomycin Solution, Corning) and fetal bovine serum (FBS, Capricorn) – 10% (cell passaging) and 2% (cell maintenance and experiments). Phosphate buffered saline (PBS) and trypsin were bought from Corning, whereas, MTT (3-(4,5-dimethylthiazol-2-yl)-2,5-diphenyltetrazolium bromide) and DMSO (dimethyl sulfoxide) from Sigma (Sigma-Aldrich, St. Louis, MO, USA). Incubation was carried out in 5% CO_2 atmosphere at 37°C (CO_2 incubator, Panasonic Healthcare Co., Tokyo, Japan).

Cytotoxicity Assessment

Cytotoxicity was tested using MTT based protocol following a previously described protocol [60]. Briefly, the cells were passaged into 96-well plates (Falcon, TC-treated, Corning) and after overnight incubation treated with serial dilutions of extract stock solutions (methanolic extracts: 1000 – 0.98 μg/mL, aqueous extracts: 2000 – 0.98 μg/mL) for 72 h. Afterwards, the media was removed, cells were washed with PBS and 10% of MTT solution (5 mg/mL) in cell media was added and the incubation continued for the next 4 h. Subsequently, the SDS/DMF/PBS (14% SDS, 36% DMF, 50% PBS) solvent was used (100 μL per well) to dis-solve the precipitated formasane crystals and the plates were left in 37°C overnight. Finally, the Synergy H1 Multi-Mode Microplate Reader (BioTek Instruments, Inc. Winooski, Vermont, USA) with Gen5 software (ver. 3.09.07; BioTek Instruments, Inc.) was used to measure the absorbance (540 and 620 nm).

Antiviral Assay

The antiviral activity of *O. aquatica* and *O. silaifolia* extracts was tested against HSV-1 (ATCC, Cat. No. VR-260) propagated in VERO cell line. The antiviral assays involved the influence of extracts on the formation of virus (HSV-1) induced cytopathic effect (CPE) and the evaluation of the reduction of infectious titer using the end-point virus titration.

The infectious titer of HSV-1 used in this study was 5.5 ± 0.25 $logCCID_{50}$/mL ($CCID_{50}$ – 50% cell culture infectious dose). Briefly, the VERO cells (monolayer) in 48-well plates (Falcon, clear flat bottom TC-treated, Corning) was treated (500 μL/well) with HSV-1 suspension (100* $CCID_{50}$/mL) in cell media and incubated for 1 h, leaving at least 2 uninfected wells as VERO cell control. Afterwards, the media were removed, monolayers washed with PBS, and the non-toxic concentrations of extracts, highest concentration not exceeding the CC_{10} values, diluted in cell media were added. The non-infected VERO cells (cell control) and non-treated infected cells (virus control) wells were maintained in media containing 2% FBS. The incubation was conducted until cytopathic effect (CPE)

was observed (inverted microscope CKX41, Olympus Corporation, Tokyo, Japan) in virus control, usually approx. 72 h. Afterwards, the plates were observed for possible inhibition of CPE by tested extracts in comparison with the CPE in virus control, and the results were recorded. Lastly, the plates were thrice frozen (−72°C) and thawed, the samples were collected, and stored at −72 °C until used in end-point virus titration assay. Antiviral properties of extracts were tested in three independent experiments.

End-Point Dilution Assay for HSV-1 Titration

Samples collected from antiviral assays were subjected to end-point dilution assay to evaluate HSV-1 titers. Briefly, the VERO cells (monolayer) in 96-well plates were incubated with ten-fold dilutions of samples (3 replicates) in cell media for 72 h. Daily observation was conducted to monitor the development of CPE. After the incubation, all media were removed and the HSV-1 infectious titer for each sample was measured using previously described MTT method. Subsequently, the difference (Δlog) of HSV-1 infectious titer ($logCCID_{50}$/mL) in the samples treated with tested *Oenanthe* extracts (OE) and in the herpesvirus control (HC) from the same experiment (Δlog = $logCCID_{50}HC$ – $logCCID_{50}OE$) were calculated. The Δlog values were evaluated for every antiviral assay and the results were expressed further as means of viral titer reduction. A significant antiviral activity can be reported for extracts decreasing the infectious titer by at least 3 log compared to virus control [60].
